# Evaluation of soybean meal bioactive peptides and Spirulina powder as antibiotic alternatives on growth performance, physiological responses, and intestinal gene expression in broilers

**DOI:** 10.1016/j.psj.2026.108020

**Published:** 2026-10-21

**Authors:** Ali Jafarzadeh, Kaveh Jafari-Khorshidi, Mohammad Ali Jafari, Shahabodin Gharahveysi, Sakineh Asadzadeh

**Affiliations:** Department of Animal Science, QaS.C., Islamic Azad University, Qaemshahr, Iran

**Keywords:** Amino acid digestibility, Antioxidant status, Digestive enzyme, Immune, Morphology

## Abstract

This study aimed to evaluate the effects of dietary soybean-derived bioactive peptides (**SBP**) and Spirulina powder (**SP**) as antibiotic alternatives on performance, amino acid digestibility, antioxidant status, and intestinal health-related gene expression in broiler chickens. A total of 250 male and female Ross 308 one-day-old chicks were used in a completely randomized design with 5 treatments, 5 replicates, and 10 chicks per replicate. The experimental treatments included: (1) a control diet consisting of the standard basal diet without any additive (CON); (2) a basal diet supplemented with avilamycin at 100 g/ton of feed (ANT); (3) a basal diet supplemented with SBP at 2 g/kg of feed (SBP); (4) a basal diet supplemented with SP at 2 g/kg of feed (SP); and (5) a basal diet supplemented with a combination of 1 g/kg SBP and 1 g/kg SP (SBP-SP). Body weight gain and feed intake during the starter and grower phases, as well as over the entire experimental period (1–42 d), was significantly greater in birds fed the SBP-SP diet compared with the CON and SP groups (P < 0.05). Broilers fed the SBP-SP, ANT, and SBP diets exhibited higher apparent ileal digestibility of Methionine, Isoleucine, Threonine, Aspartic acid and Glutamic acid compared with those fed the CON and SP diets (P < 0.05). Dietary inclusion of SBP-SP and ANT led to a substantial increase in both villus height, villus surface area, and the villus height to crypt depth ratio when compared to the CON group. Dietary supplementation with SP and SBP-SP significantly boosted the IBV antibody titers at both 21 d (P = 0.006) and 42 d (P = 0.017) compared to the CON, ANT, and SBP groups. The relative weight of the Bursa of Fabricius and Thymus was markedly increased in birds supplemented with SBP-SP and SP compared to those in the CON and SBP groups (P = 0.015). Broilers receiving the SBP-SP and SP treatments exhibited significantly higher levels of TAC, SOD, GPx, and CAT compared with the CON and ANT groups. The mRNA levels of *IL-1β* and *TNF-α* were markedly downregulated in birds receiving the SBP-SP diet compared to all other experimental groups (P < 0.05). The expression of *ZO-1, MUC2, SLC15A1, SLC6A19* and *SLC7A1* was significantly upregulated in the SBP-SP group compared to the CON group (P < 0.05). Collectively, these findings indicate that the combined use of SBP and SP exerts synergistic effects on digestive efficiency, intestinal function, and physiological status, suggesting that this combination may serve as a promising natural alternative to antibiotic growth promoters in broiler nutrition.

## Introduction

The global poultry sector faces mounting pressure to curtail or phase out the routine administration of antibiotic growth promoters, driven by escalating concerns over antimicrobial resistance, residual drug traces in animal products, and rising consumer demand for antibiotic-free poultry (Hedman et al., 2020). Although antibiotics have historically been used to improve growth performance and control enteric disorders in broiler chickens, their restrictions in many countries have intensified the search for safe, effective, and sustainable nutritional alternatives (Tian et al., 2021). In this context, bioactive feed additives capable of improving nutrient utilization, enhancing immune competence, maintaining intestinal barrier integrity, and modulating oxidative stress have received considerable attention in broiler nutrition (Abd El-Aziz et al., 2025; Lu et al., 2026). Soybean-derived bioactive peptides (**SBP**) have emerged as promising functional ingredients in animal feeding (Mahgoub et al., 2023; Singh et al., 2023). Bioactive peptides—typically liberated through the enzymatic hydrolysis or fermentation of dietary proteins—have been shown to elicit a broad spectrum of physiological activities, encompassing antioxidant and immunomodulatory functions, as well as the regulation of nutrient transport (Hou et al., 2022). Soybean meal, as one of the most widely used plant protein sources in poultry diets, contains a diverse range of encrypted peptide sequences that can be liberated during hydrolysis (Pope et al., 2023). Compared with intact proteins, low-molecular-weight peptides may be more rapidly absorbed through peptide transport systems, particularly peptide transporter 1, and may enhance amino acid availability and nitrogen utilization (Mohammadrezaei et al., 2025). Concurrently, microalgal biomass such as Spirulina platensis (**SP**), rich in valuable bioactive phytocompounds, has emerged as another potent candidate to serve alongside functional peptides as natural antibiotic growth promoter alternatives (Hajati et al., 2024).

Spirulina platensis, a filamentous cyanobacterium distinguished by its abundance of high-quality protein, amino acids, pigments, polysaccharides, vitamins, and bioactive constituents, has also attracted interest as a natural growth-promoting and health-enhancing feed additive (Abd El-Hady et al., 2022). Earlier investigations have indicated that dietary supplementation with SP can enhance performance, strengthen humoral immune responses, increase antioxidant capacity, and beneficially modulate gut microbial populations in broiler chickens (Awad et al., 2023; Hajati et al., 2024; Spínola et al., 2024). Although plant-derived bioactive peptides and SP have each shown beneficial effects on poultry health and productivity (Alghamdi et al., 2024; Peng et al., 2025), their combined application remains underexplored. Theoretically, these two bioactive ingredients may act through complementary mechanisms. Plant-derived peptides may directly enhance amino acid absorption, stimulate digestive enzyme activity, and regulate the expression of genes involved in nutrient transport and intestinal barrier function (Xie et al., 2020). In contrast, SP may provide additional antioxidant, immunostimulatory, and prebiotic-like effects through its pigments, polysaccharides, and micronutrients (Mirzaie et al., 2017).

While individual benefits of bioactive peptides and microalgal biomass are recognized, whether their concurrent supplementation confers complementary or synergistic benefits remains an unexplored question. Mechanistically, SBP primarily facilitate mucosal nutrient uptake, stimulate digestive secretions, and upregulate amino acid transporter expressions, whereas SP is uniquely positioned to enhance systemic antioxidant defenses, fortify mucosal immune status, and support barrier integrity. We hypothesized that combining these two distinct nutritional bioactives would provide a multifaceted mode of action—simultaneously optimizing intestinal absorptive capacity and dampening enteric oxidative and immune stressors—surpassing the efficacy of single-additive regimes or antibiotic growth promoters. Therefore, the present study was designed to evaluate the individual and combined effects of SBP and SP powder on growth performance, nutrient digestibility, digestive enzyme activity, antioxidant-immune status, and the expression of intestinal barrier and nutrient transporter genes in broiler chickens.

## Methods and materials

### Animal ethics

All animal-based experimental procedures conducted in this study received prior review and formal approval from the Animal Care and Animal Research Ethics Committee of the Islamic Azad University, Sari Branch, Iran (Approval No. IR.IAU.SARI.REC.1404.029).

### Birds, treatments, and management

A total of 250 one-day-old Ross 308 broiler chicks of both sexes, with an average initial body weight of 44.7 ± 0.38 g, were randomly allocated in a completely randomized design comprising 5 experimental treatments, each with 5 replicates of 10 birds (5 males and 5 females). The experimental treatments included: (1) a control diet consisting of the standard basal diet without any additive (CON); (2) a basal diet supplemented with avilamycin at 100 g/ton of feed (Avilamycin 10%, Rooyan Darou Co., Tehran, Iran) (ANT); (3) a basal diet supplemented with SBP at 2 g/kg of feed (SBP); (4) a basal diet supplemented with SP at 2 g/kg of feed (SP); and (5) a basal diet supplemented with a combination of SBP at 1 g/kg of feed and SP at 1 g/kg of feed (SBP-SP). The SBP and SP were supplied by Tak Gene Knowledge-Based Company, Tehran, Iran. Birds were reared on floor pens measuring 100 cm (length) × 70 cm (width) × 60 cm (height). Throughout the trial, a photoperiod of 23 h light and 1 h dark (23L:1D) was applied during the first week of age, which was subsequently adjusted to 18L:6D for the remainder of the experimental period. Relative humidity was maintained at approximately 50%, while ambient temperature was regulated according to the age-specific recommendations outlined in the Ross 308 Broiler Management Handbook (2022, Aviagen). Isocaloric and isonitrogenous experimental diets were formulated for the starter (1–10 d), grower (11–24 d), and finisher (25–42 d) phases, as detailed in Table 1. Throughout the study, feed and fresh water were provided ad libitum.

**Table 1:** Components and chemical compositions of the diets during starter, grower and finisher periods (as-is basis).

Ingredients %	Starter (d 1-10)	Grower (d 11-24)	Finisher (d 25-42)
Corn	55.12	57.30	62.41
Soybean meal (44%)	39.28	36.31	30.98
Soybean oil	1.30	2.26	2.75
Dicalcium phosphate	1.55	1.44	1.30
Calcium carbonate	1.10	1.00	0.96
DL-Met	0.32	0.30	0.27
L-Lys HCl	0.24	0.18	0.19
L-Thr	0.10	0.06	0.04
Vitamin premix^1^	0.25	0.25	0.25
Mineral premix^2^	0.25	0.25	0.25
Salt	0.29	0.45	0.40
Sodium bicarbonate	0.20	0.20	0.20
**Calculated composition**			
ME, Kcal/Kg	2800	2900	3000
Crude protein, %	21.50	20.50	18.50
Crude fiber, %	3.96	3.80	3.54
Linoleic acid, %	2.03	2.56	2.90
Calcium, %	0.90	0.82	0.76
Available phosphorus, %	0.45	0.42	0.39
Sodium, %	0.17	0.18	0.18
Chloride, %	0.20	0.20	0.20
Dig. lysine, %	1.32	1.19	1.00
Dig. methionine, %	0.62	0.58	0.53
Dig. methionine + cysteine, %	0.92	0.86	0.80
Dig. threonine, %	0.82	0.74	0.65
**Analyzed value**			
Gross energy (kcal/kg)	4108	4257	4316
Crude protein (%)	21.52	20.53	18.49
Calcium (%)	0.90	0.81	0.78
Total phosphorus (%)	0.71	0.67	0.62

1 Provides the following per kg of diet: 4.13 mg retinol, 60.00 µg chole-calciferol, 30.00 mg Dl-α-tocopherol, 3mg menadione, 2.20 mg thiamine, 8.00 mg riboflavin, 5.00 mg pyridoxine, 11.00 µg cyanocobalamin, 1.50 mg folic acid, 150.00 µg biotin, 25.00 mg calcium pantotenate, 65.00 mg nicotinic acid. Provides the following per kg of diet: 60.00 mg Mn (manganese sulphate), 40.00 mg Zn (zinc oxide), 0.33 mg I (potassium iodate), 80.00 mg Fe (ferrous sulphate), 8.00 mg Cu (copper sulphate), 0.15 mg Se (sodium selenite), 150.00 mg ethoxyquin.

### Performance

Feed intake (**FI**) and growth-related variables were evaluated on a pen basis throughout the 42-day trial. Before the daily feed allocation, the quantity of feed supplied to each experimental unit was weighed and recorded. At the end of each feeding phase, the residual feed was weighed, and phase-specific feed intake was calculated as the difference between the cumulative feed offered and the feed remaining. The same procedure was used to determine body weight gain (**BWG**), which was calculated for each phase by subtracting the mean body weight recorded at the beginning of the phase from that measured at its completion. To assess live body weight, all chicks belonging to the same replicate were weighed together on days 1, 10, 24, and 42. Feed conversion ratio (**FCR**) was determined independently for the starter, grower, and finisher periods by relating mean FI to the corresponding mean BWG. Mortality was checked in every pen each day before feed distribution. Any deceased bird was weighed immediately, and the resulting mortality records were used to calculate the chicken-day value for each replicate according to the method described by Ebrahimi et al., 2026.

### Apparent ileal protein and amino acid digestibility

To determine the apparent ileal digestibility of crude protein and amino acids, two male broilers per replicate were randomly selected and slaughtered at the end of the trial. Ileal digesta were obtained by gently flushing the segment extending from approximately 20 mm proximal to the ileo-cecal junction to the distal ileum, applying slight pressure to expel the contents. The collected samples were placed in labeled plastic containers and immediately stored at −20°C pending laboratory analysis. Crude protein concentrations in both diets and ileal digesta were calculated as nitrogen percentage × 6.25, with nitrogen quantified using the Micro-Kjeldahl method. The apparent ileal digestibility of crude protein and amino acids was determined using the following equation (Ravindran et al., 2005):

$$\text{AID}\;\left(\%\right)=\left[1-\left(\frac{AIA_{d}\;}{AIA_{i\;}}\;\times \frac{N_{i}\;}{N_{d\;}}\right)\right]\times \;100$$

where AIA_d_​ and N_d_​ represent the concentrations of acid-insoluble ash (AIA) and nutrient (crude protein or amino acid) in the diet, respectively, and AIA_i_​ and N_i_ denote the corresponding concentrations in the ileal digesta.

The quantification of amino acids in the ileal digesta was carried out following the protocol outlined by Zafar et al. (2025), employing an automated analyzer (Biochrom 30+, Biochrom Limited, Cambridge, UK). In brief, samples were first milled to a particle size not exceeding 0.5 mm. To protect the sulfur-bearing residues from oxidative loss, samples underwent oxidation with performic acid, which converted methionine into methionine sulfone and cysteine into cysteic acid. This step was followed by acid hydrolysis in 6 M hydrochloric acid supplemented with phenol for a 24-hour period. Once hydrolysis was complete, the resulting solutions were neutralized to pH 2.2, clarified by filtration, and dispensed into vials ready for analysis. Separation and detection of the individual amino acids were then achieved through ion-exchange chromatography on the Biochrom 30+ system.

### Digestive enzyme activity

On day 42, jejunal digesta samples were obtained from two male birds per replicate. A subsample (0.1 g) was homogenized with 900 µL of sterile saline solution and kept at 4°C. The homogenates were vortexed thoroughly and centrifuged at 3,000 rpm for 10 minutes to separate the supernatant. Using this supernatant, the enzymatic activities of amylase, lipase, and total protease were measured with chicken-specific ELISA kits (Delta Darman Part Co., Tehran, Iran), strictly adhering to the manufacturer’s protocol (Guo et al., 2025).

### Jejunum morphology

Morphometric evaluation of the jejunum was performed at 42 days of age, when two male birds per replicate were sacrificed to harvest tissue for histological analysis, in accordance with the protocol of Khalilzadeh et al. (2025). A jejunal segment of roughly 1 cm in length was removed and washed gently with 0.9% saline to clear any residual luminal contents. To arrest post-mortem degradation, the specimens were immediately immersed in 10% buffered formalin. Standard tissue processing then followed, entailing sequential dehydration, clearing, and paraffin embedding. Serial sections of 7 μm thickness were cut using a microtome (Microm, HM 335) and affixed onto glass slides. Before staining, paraffin was removed by immersion in xylene, after which the sections were progressively rehydrated through descending concentrations of ethanol. Hematoxylin and eosin (H&E) staining was subsequently applied, and the slides were examined under a light microscope. The intestinal architecture was quantified in terms of villus height, villus width, crypt depth, and villus surface area. Villus height was recorded as the distance spanning from the villus apex to the upper limit of the lamina propria, whereas crypt depth was measured from the villus–crypt junction down to the muscularis mucosa. The villus-height-to-crypt-depth ratio (VH/CD) was derived by dividing the former measurement by the latter (Hassanlou et al., 2026). Villus surface area was additionally estimated using the equation (2π) × (villus width × 0.5) × villus length (Sakamoto et al., 2000).

### Determination of antibody titers

Broiler chickens were vaccinated against infectious bronchitis virus (**IBV**) and infectious bursal disease virus (**IBDV**) according to the standard regional vaccination program. Birds received the first vaccination at 1 day of age via spray administration, followed by a booster vaccination at 10 days of age via subcutaneous injection in the neck region using commercially available inactivated vaccines (Rooyan Darou Co., Tehran, Iran). To evaluate the humoral immune response, blood samples were collected from two randomly selected male birds per replicate at 21 and 42 days of age. Birds selected for blood sampling had body weights close to the replicate mean to minimize sampling bias. Blood samples (2-3 mL) were drawn from the wing vein of each bird using sterile syringes and immediately dispensed into anticoagulant-free vacuum tubes. Clotting was permitted to proceed at ambient temperature over a 30-60-minute window, after which the tubes were centrifuged at 3,000 rpm for 10 minutes under refrigerated conditions (4°C) to separate the serum fraction. The clarified serum was then aspirated with care and aliquoted into sterile microtubes for long-term storage at −20°C pending subsequent assays. Serum antibody responses directed against IBV and IBDV were quantified by enzyme-linked immunosorbent assay using commercial kits (BioCheck B.V., Reeuwijk, Netherlands), with all procedures executed according to the manufacturer’s specifications.

### Immune organ indices

At the end of the trial, two male birds from each replicate were individually weighed and slaughtered according to approved ethical procedures. Immediately after slaughter, the spleen, thymus, and bursa of Fabricius were carefully removed. Adhering fat and connective tissues were trimmed, and organs were gently blotted dry using filter paper. Each organ was weighed using a digital balance (0.01 g; KEB 602, China). The relative weight of immune organs was calculated as g/kg of live body weight using the following equation (Xie et al., 2020):

$$\text{Immune}\;\text{organ}\;\text{index}\;\left(g/\text{kg}\right)=\frac{\text{Organ}\;\text{weight}\;\left(g\right)}{\text{Live}\;\text{body}\;\text{weight}\;\left(\text{kg}\right)}$$

The spleen, thymus, and bursa of Fabricius were evaluated as indicators of immune organ development and immune competence.

### Oxidative stress and antioxidant indices

Oxidative balance was evaluated at the conclusion of the trial, when birds reached 42 days of age. Following a 4-h fasting period, blood was obtained from the wing vein of two male birds within each replicate. Samples were kept at room temperature until clot formation was complete and subsequently centrifuged at 3,000 × *g* for 15 min at 4°C. Only non-hemolyzed serum samples were retained, transferred to appropriate storage tubes, and frozen at −20°C until biochemical analyses were conducted. Serum concentrations of malondialdehyde (**MDA**) and total antioxidant capacity (**TAC**), together with the activities of superoxide dismutase (**SOD**), glutathione peroxidase (**GPx**), and catalase (**CAT**), were determined colorimetrically using commercial ZellBio assay kits (ZellBio GmbH Co., Ulm, Germany). All determinations followed the kit manufacturer’s prescribed procedures and were performed in duplicate. Results were expressed as nmol/mL for MDA, U/mL for SOD and GPx, and mmol/L for TAC and CAT. Analytical precision was verified through intra- and inter-assay coefficients of variation. For MDA, the respective intra- and inter-assay CVs were 5.8% and 7.6%. TAC showed intra-assay variability between 2.2% and 3.0%, whereas inter-assay variability ranged from 2.9% to 4.2%. The corresponding ranges for SOD were 4.6–16.8% within assays and 6.1–13.8% between assays. For GPx, intra-assay CVs varied from 2.1% to 5.0%, while inter-assay CVs ranged from 7.5% to 13.3%.

### Gene expression analysis

At 42 days of age, intestinal tissue was sampled from 10 birds within each dietary treatment. These birds represented two randomly selected male birds from each cage that were humanely euthanized by carbon dioxide inhalation. For molecular analyses, a 2-cm portion of the middle jejunum, anatomically located between the terminal region of the duodenal loop and Meckel’s diverticulum, was excised. Luminal residues were removed by rinsing the segments with ice-cold phosphate-buffered saline (PBS). The mucosal layer was then carefully collected by scraping the inner intestinal surface with a sterile glass slide. Samples were snap-frozen in liquid nitrogen immediately after collection and maintained at −80°C until RNA isolation, following Al Amaz et al., 2025. Transcriptional responses were evaluated for genes representing intestinal inflammation, epithelial barrier integrity, mucin production, and nutrient absorption. The inflammatory panel included *IL-1β, IL-6*, and *TNF-α* as pro-inflammatory markers, together with *IL-10* as an anti-inflammatory marker. The expression of *Occludin, Claudin-1*, and *ZO-1* was assessed to characterize tight-junction function, whereas *MUC2* was selected as an indicator of mucin-associated epithelial protection. In addition, *SLC15A1, SLC6A19, SLC7A1*, and *SLC3A1* were examined as genes involved in intestinal nutrient transport.

Total RNA was isolated from jejunal mucosal tissue using TRIzol reagent (Invitrogen, Carlsbad, CA, USA). A 1-µg aliquot of extracted RNA was reverse-transcribed to complementary DNA using the Maxima First Strand cDNA Synthesis Kit (Life Technologies, Carlsbad, CA, USA). The primers used for amplification are listed in Table 2. Quantitative real-time PCR was carried out on a 7500 Fast Real-Time PCR System (Applied Biosystems, Foster City, CA, USA), following the manufacturer’s recommended procedure. Amplification included denaturation at 94°C for 30 s, primer annealing at 59–61°C for 30 s, and extension at 72°C for 30 s. Before the final expression analysis, melting-curve profiles were inspected to verify the generation of a single, specific amplification product for every target. Expression values were normalized against *β-actin*, and relative transcript abundance was calculated according to the 2^−ΔΔCt^ method described by Livak and Schmittgen, 2001.

**Table 2:** Primers used in gene quantification by qPCR.

**Gene**	**Forward Primer (5′−3′)**	**Reverse Primer (5′−3′)**
*β-actin*	TGCTGTGTTCCCATCTATCG	TTGGTGACAATACCGTGTTCA
**Immune**		
*IL-1β*	GGAGAAGGACCCCGAATACTG	TTGACGCCGCAGAGGTAAG
*IL-6*	TTCACAAGCATTTCATCCCTC	TTGCGGACATACCCAGTGACA
*TNF-α*	CCAGCACCTCGAGCAAT	GGCGCTGTAATCGTTGTCT
*IL-10*	CACGCGGAGGGCGTTAAA	CAGGTGAAAGTCAGCCCGT
**Intestinal Barrier**		
*Occludin*	GTGTAAGGCCCACACCTCTG	TGAGCTGTGTGCTCAGGGTA
*Claudin-1*	TGGAGGATGACCAGGTGAAGA	CGAGCCACTCTGTTGCCATA
*ZO-1*	GCCTGAATCAAACCCAGCAA	TATGCGGCGGTAAGGATGAT
*MUC2*	TTCATGATGCCTGCTCTTGTC	CCGTAGCCTTGGTACATTCTTGT
**Nutrient Transporter**		
*SLC3A1*	GGCTTACCAATGGAGCTGAGTGAG	CTTGGCTGCTGGTGTCAGTATCC
*SLC6A19*	CTCCAGTGGGCTGGATGAGA	CAAGGCAGCTCCAACGATCA
*SLC15A1*	CCTGTCACCTTCCGCTGTGC	GCTGCTGTTGCTGTGACACTAATG
*SLC7A1*	GGAGCCTTGTGGGTGTGTAA	CTAGGGGAACGCTGCTAAGT

### Statistical analysis

Before analysis, the normality of all collected data was assessed using the PROC UNIVARIATE procedure in SAS (version 9.2). Percentage-based variables failing to meet distributional assumptions were subjected to ArcSin√x transformation before modeling. All experimental data were subsequently evaluated by analysis of variance implemented through the PROC GLM procedure in SAS (version 9.2). Multiple comparisons among treatment means were performed using Tukey’s test, with statistical significance declared at P<0.05. The underlying statistical model employed in this investigation was defined as:

$$Y_{ij}=\mu +T_{i}+\varepsilon_{ij}$$

where *Y_ij_* denotes the individual observed value, *μ* represents the overall mean, *Ti*​ corresponds to the fixed effect of the i th treatment, and *ε_ij_*​ is the residual error associated with each observation.

## Results

### Broiler performance

The effects of dietary treatments on broiler growth performance are presented in Table 3. Average feed intake was significantly higher in the SBP-SP group compared with the CON and SP treatments throughout all experimental periods (P < 0.05). Additionally, broilers fed ANT and SBP diets exhibited greater feed intake than the CON group during the starter (1–10 d) and grower (11–24 d) phases (P < 0.05). Body weight gain during the starter and grower phases, as well as over the entire experimental period (1–42 d), was significantly greater in birds fed the SBP-SP diet compared with the CON and SP groups (P < 0.05); however, no significant differences were observed among treatments during the finisher phase (25–42 d, P = 0.205). Regarding the feed conversion ratio, a significant dietary effect was only observed during the grower phase (11–24 d), where broilers fed the SBP-SP treatment exhibited a lower FCR compared with birds in the CON and SP groups (P = 0.004). Overall, the combined SBP-SP treatment consistently elicited superior growth responses and feed efficiency, particularly during the earlier rearing phases, outperforming individual supplementations.

**Table 3:** The effect of soybean meal bioactive peptides and Spirulina powder on the performance of broiler chickens (50 birds per treatment).

Items	CON	ANT	SBP	SP	SBP-SP	SEM	*P*-value
**Average feed intake, g/d**							
1-10d	31.42^c^	35.41^ab^	35.89^ab^	34.12^b^	36.69^a^	0.68	0.018
11-24d	82.14^c^	85.97^ab^	85.60^ab^	84.43^b^	87.57^a^	0.82	0.034
25-42d	154.39^b^	164.50^ab^	165.13^ab^	156.56^b^	169.13^a^	1.87	0.021
1-42d	100.83^b^	106.79^ab^	107.88^ab^	101.65^b^	110.41^a^	1.39	0.008
**Body weight gain, g/d**							
1-10d	27.65^b^	28.79^ab^	28.85^ab^	28.35^ab^	29.48^a^	0.48	0.007
11-24d	55.97^c^	62.85^b^	61.71^b^	57.72^bc^	65.78^a^	1.07	0.023
25-42d	83.87	85.65	85.76	83.71	86.37	1.65	0.205
1-42d	61.19^b^	63.75^ab^	62.19^ab^	62.29^b^	65.96^a^	0.86	0.002
**Feed conversion ratio**							
1-10d	1.22	1.23	1.27	1.26	1.26	0.05	0.462
11-24d	1.50^a^	1.39^ab^	1.40^ab^	1.48^a^	1.35^b^	0.04	0.004
25-42d	1.86	1.97	1.95	1.93	1.98	0.09	0.326
1-42d	1.67	1.68	1.70	1.72	1.70	0.07	0.218
Initial weight, g	44.62	44.78	44.65	44.81	44.63	0.38	0.862
Final body weight, g	2614.60^b^	2722.28^ab^	2656.63^b^	2660.99^b^	2814.95^a^	34.09	0.014
Mortality, %	2.00	4.00	2.00	2.00	4.00	0.43	0.185

CON = basal diet; ANT = basal diet + antibiotic; SBP= basal diet + soybean-derived bioactive peptides; SP = basal diet + Spirulina powder; SBP-SP = basal diet + soybean-derived bioactive peptides + Spirulina powder. SEM: standard error of the mean.

^a-b^ Means within a variable with no common superscript differ significantly (P<0.05).

### Protein and amino acid digestibility

Table 4 presents the effects of SBP and SP on the apparent ileal digestibility of crude protein and amino acids in broiler chickens. The digestibility of crude protein was significantly improved in birds receiving SBP-SP, SBP, and ANT diets compared with the CON group (P = 0.014). Regarding essential amino acids, dietary treatments significantly influenced the digestibility of Methionine, Lysine, Leucine, Isoleucine, Threonine, and Arginine (P < 0.05). Specifically, broilers fed the SBP-SP, ANT, and SBP diets exhibited higher apparent ileal digestibility of Methionine, Isoleucine, and Threonine compared with those fed the CON and SP diets (P < 0.05). The digestibility of Leucine (P = 0.026) and Arginine (P = 0.003) was highest in the SBP-SP and ANT groups, showing a significant increase over the CON and SP treatments. However, no significant differences were observed for Valine (P = 0.218), Histidine (P = 0.361), and Phenylalanine (P = 0.548) among the experimental groups. For non-essential amino acids, the apparent ileal digestibility of Aspartic acid (P = 0.038) and Glutamic acid (P = 006) was significantly enhanced in the SBP-SP, SBP, and ANT groups compared with the CON and SP treatments. Collectively, dietary SBP, either alone or combined with SP, was the principal driver of improved ileal protein and amino acid digestibility, demonstrating an efficacy comparable to the antibiotic control.

**Table 4:** The effect of soybean meal bioactive peptides and Spirulina powder on apparent ileal digestibility of crude protein and amino acids of broiler chickens (10 birds per treatment).

Items	CON	ANT	SBP	SP	SBP-SP	SEM	*P*-value
Crude protein	79.30^b^	82.47^a^	82.65^a^	81.13^ab^	82.98^a^	0.81	0.014
**Essential amino acids**							
Methionine	84.89^b^	89.85^a^	89.31^a^	88.00^ab^	90.66^a^	0.89	0.007
Lysine	83.24^b^	90.88^a^	89.53^a^	84.93^ab^	91.30^b^	1.23	0.034
Leucine	77.38^b^	81.62^a^	80.89^ab^	79.18^b^	82.88^a^	1.18	0.026
Isoleucine	72.95^b^	77.43^a^	78.42^a^	74.21^b^	79.55^a^	1.27	0.009
Threonine	66.02^b^	69.07^a^	68.40^a^	65.74^b^	68.32^a^	0.92	0.048
Valine	65.03	66.78	66.19	65.29	67.38	0.84	0.218
Histidine	85.52	86.21	86.30	85.22	86.53	0.82	0.361
Arginine	85.91^b^	92.50^a^	90.63^ab^	86.62^b^	92.06^a^	1.42	0.003
Phenylalanine	78.66	79.09	79.61	78.52	79.95	1.53	0.548
**Non-essential amino acids**							
Glycine	68.82	69.55	68.34	67.16	70.68	1.34	0.275
Alanine	77.21	78.56	78.13	76.32	78.27	1.21	0.139
Aspartic acid	81.65^b^	86.59^a^	86.94^a^	81.38^b^	87.93^a^	1.29	0.038
Cysteic acid	85.56	85.91	85.03	84.37	86.16	1.01	0.261
Serine	79.96	79.65	79.52	79.31	80.29	0.91	0.534
Glutamic acid	84.21^b^	88.98^a^	87.99^a^	83.03^b^	88.60^a^	0.87	0.006
Tyrosine	81.18	82.85	81.90	82.38	83.05	1.11	0.495
Ornithine	78.79	79.18	78.13	77.32	79.36	1.19	0.365
Proline	80.81	80.69	80.88	79.17	80.10	1.25	0.108

CON = basal diet; ANT = basal diet + antibiotic; SBP= basal diet + soybean-derived bioactive peptides; SP = basal diet + Spirulina powder; SBP-SP = basal diet + soybean-derived bioactive peptides + Spirulina powder. SEM: standard error of the mean.

^a-b^ Means within a variable with no common superscript differ significantly (P<0.05).

### Digestive enzyme activities

The effect of dietary treatments on the digestive enzyme activities of broiler chickens is summarized in Table 5. Activities of Amylase and Total protease were significantly influenced by the dietary supplementations (P < 0.05). Specifically, broilers fed the ANT, SBP, SP, and SBP-SP diets exhibited significantly higher activities of Amylase (P = 0.012) and Total protease (P = 0.007) compared with those receiving the CON diet. However, no significant differences were observed among the experimental groups regarding Lipase activity (P = 0.538). In general, both SBP and SP exerted stimulatory effects on proteolytic and amylolytic pancreatic enzyme activities without modifying lipolytic secretion.

**Table 5:** The effect of soybean meal bioactive peptides and Spirulina powder on digestive enzyme activity of broiler chickens (10 birds per treatment).

Items	CON	ANT	SBP	SP	SBP-SP	SEM	*P*-value
Amylase, IU/L	483.42^b^	546.77^a^	563.19^a^	559.78^a^	555.21^a^	20.50	0.012
Lipase, U/L	1711.56	1730.03	1721.12	1740.64	1751.48	37.15	0.538
Total protease, U/L	1798.02^b^	1895.01^a^	1902.34^a^	1887.12^a^	1924.20^a^	41.96	0.007

CON = basal diet; ANT = basal diet + antibiotic; SBP= basal diet + soybean-derived bioactive peptides; SP = basal diet + Spirulina powder; SBP-SP = basal diet + soybean-derived bioactive peptides + Spirulina powder. SEM: standard error of the mean.

^a-b^ Means within a variable with no common superscript differ significantly (P<0.05).

### Jejunum morphology

Data regarding the histomorphological changes in the jejunum are detailed in Table 6. Dietary inclusion of SBP-SP and ANT led to a substantial increase in both villus height (P = 0.041) and the villus height to crypt depth ratio (P = 0.035) when compared to the CON group. A similar trend was observed for the villus surface area, which was markedly enhanced in birds receiving SBP-SP and ANT treatments, reaching significantly higher values than those recorded for the CON, SBP, and SP groups (P = 0.029). Among the supplemented groups, birds fed the SP diet also exhibited a greater villus surface area in comparison to the CON group (P = 0.029). In contrast, other morphological parameters, including villus width (P = 0.199) and crypt depth (P = 0.051), remained statistically unaffected by the various dietary treatments. These findings demonstrate that combined SBP and SP supplementation produces beneficial structural modifications in the jejunum that resemble or match the mucosal improvements achieved with antibiotic supplementation.

**Table 6:** Effect of soybean meal bioactive peptides and Spirulina powder on jejunum morphology of broiler chickens (10 birds per treatment).

Items	CON	ANT	SBP	SP	SBP-SP	SEM	*P*-value
Villus height (µm)	1006.15^b^	1169.58^a^	1060.34^ab^	1085.86^ab^	1112.49^a^	20.53	0.041
Villus width (µm)	120.93	142.10	125.73	135.10	145.33	8.49	0.199
Crypt depth (µm)	180.57	156.90	175.43	170.30	155.33	7.70	0.051
Villus height /crypt depth	5.61^b^	7.53^a^	6.04^ab^	6.39^ab^	7.44^a^	0.38	0.035
Villus surface area (µm²)	383918^c^	521971^a^	417046^bc^	457698^b^	508016^a^	905.6	0.029

CON = basal diet; ANT = basal diet + antibiotic; SBP= basal diet + soybean-derived bioactive peptides; SP = basal diet + Spirulina powder; SBP-SP = basal diet + soybean-derived bioactive peptides + Spirulina powder. SEM: standard error of the mean.

^a-b^ Means within a variable with no common superscript differ significantly (P<0.05).

### Immune response and immune organ indices

The humoral immune response, evaluated through antibody titers against IBV and IBDV, is presented in Table 7. Dietary supplementation with SP and SBP-SP significantly boosted the IBV antibody titers at both 21 d (P = 0.006) and 42 d (P = 0.017) compared to the CON, ANT, and SBP groups. A similar stimulatory effect was observed for IBDV antibody titers at 21 d, where birds receiving SP and SBP-SP exhibited significantly higher titers than those in the other experimental groups (P = 0.023). However, by the end of the trial (42 d), no significant differences were detected in IBDV antibody levels among any of the dietary treatments (P = 0.294). Interestingly, the inclusion of ANT or SBP did not result in a significant elevation of antibody titers against either virus when compared to the control group throughout the study.

**Table 7:** Effect of soybean meal bioactive peptides and Spirulina powder on antibody titers and immune organ index of broiler chickens (10 birds per treatment).

Items	CON	ANT	SBP	SP	SBP-SP	SEM	*P*-value
**IBV antibody titer**							
21 d	5.46^b^	5.67^b^	5.53^b^	6.27^a^	6.39^a^	0.16	0.006
42 d	4.13^b^	4.25^b^	4.18^b^	5.38^a^	5.44^a^	0.29	0.017
**IBDV antibody titer**							
21 d	5.08^b^	5.16^b^	5.27^b^	5.94^a^	5.87^a^	0.18	0.023
42 d	4.27	4.35	4.38	4.49	4.53	0.26	0.294
**Immune organ index**							
Spleen (% of BW)	1.79	1.63	1.65	1.52	1.50	0.19	0.107
Bursal (% of BW)	1.16^b^	1.84^a^	1.28^b^	1.95^a^	2.06^a^	0.17	0.015
Thymus (% of BW)	2.05^b^	2.68^ab^	2.11^b^	3.02^a^	3.17^a^	0.24	0.006

CON = basal diet; ANT = basal diet + antibiotic; SBP= basal diet + soybean-derived bioactive peptides; SP = basal diet + Spirulina powder; SBP-SP = basal diet + soybean-derived bioactive peptides + Spirulina powder. SEM: standard error of the mean.

^a-b^ Means within a variable with no common superscript differ significantly (P<0.05).

The relative weights of immune organs as a percentage of body weight are illustrated in Table 7. Dietary interventions significantly altered the Bursal and Thymus indices (P < 0.05). Specifically, the relative weight of the Bursa of Fabricius was markedly increased in birds supplemented with SBP-SP, SP, and ANT compared to those in the CON and SBP groups (P = 0.015). Similarly, the Thymus index was significantly enhanced by the SBP-SP and SP treatments relative to the CON and SBP diets (P = 0.006), while the ANT group showed intermediate values. In contrast, the relative weight of the Spleen remained unaffected by any of the dietary treatments, with no significant differences observed among the experimental groups (P = 0.107). Taken together, treatments containing SP (alone or with SBP) served as the primary immunostimulants, driving both humoral antibody titers and central lymphoid organ enlargement.

### Oxidative stress and antioxidant indices

The serum oxidative stress biomarkers and antioxidant enzyme activities are summarized in Table 8. A notable reduction in serum MDA levels was observed in birds fed SBP, SP, and SBP-SP diets compared with those in the CON and ANT groups (P = 0.002). Concurrently, the antioxidant capacity was enhanced by the experimental diets. Broilers receiving the SBP-SP and SP treatments exhibited significantly higher levels of TAC (P = 0.025), GPx (P = 0.003), and CAT (P = 0.018) compared with the CON and ANT groups. Furthermore, the activity of SOD was significantly elevated in all groups supplemented with SBP and/or SP (SBP, SP, and SBP-SP) relative to the CON and ANT treatments (P = 0.016). In summary, dietary inclusion of SBP and SP effectively fortified the systemic enzymatic antioxidant network while simultaneously reducing lipid peroxidation.

**Table 8:** Effect of soybean meal bioactive peptides and Spirulina powder on oxidative stress and antioxidant indices of broiler chickens (10 birds per treatment).

Items	CON	ANT	SBP	SP	SBP-SP	SEM	*P*-value
MDA (nmol/mL)	3.17^a^	2.94^a^	2.21^b^	2.06^b^	2.10^b^	0.29	0.002
TAC (mmol/L)	1.19^b^	1.58^b^	2.07^ab^	2.30^a^	2.41^a^	0.18	0.025
SOD (U/mL)	30.38^b^	33.24^b^	39.75^a^	41.81^a^	42.23^a^	1.75	0.016
GPx (U/mL)	9.64^b^	9.73^b^	10.67^ab^	12.09^a^	11.89^a^	0.54	0.003
CAT (U/mL)	4.61^b^	4.52^b^	5.09^ab^	6.12^a^	6.87^a^	0.41	0.018

CON = basal diet; ANT = basal diet + antibiotic; SBP= basal diet + soybean-derived bioactive peptides; SP = basal diet + Spirulina powder; SBP-SP = basal diet + soybean-derived bioactive peptides + Spirulina powder. MDA: Malondialdehyde; TAC: Total Antioxidant Capacity; SOD: Superoxide Dismutase; GPx: Glutathione Peroxidase; CAT: Catalase; SEM: standard error of means.

^a-b^ Means within a variable with no common superscript differ significantly (P<0.05).

### Immune gene expression

The mRNA expression levels of inflammatory-related genes in the jejunal mucosa are illustrated in Fig. 1. Specifically, the mRNA levels of *IL-1β* and *TNF-α* were markedly downregulated in birds receiving the SBP-SP diet compared to all other experimental groups. Moreover, broilers in the ANT, SBP, and SP groups exhibited lower expression of these pro-inflammatory markers relative to the CON group (P < 0.05). Conversely, the expression of the anti-inflammatory cytokine *IL-10* was significantly upregulated in the SBP-SP and ANT groups compared with the CON, SBP, and SP treatments (P = 0.001). Among the individual supplements, birds fed SBP and SP also showed higher *IL-10* mRNA levels than those in the CON group (P = 0.001). In contrast, the expression of *IL-6* remained statistically similar across all dietary treatments, with no significant differences observed among the groups (P = 0.196). Overall, the combination of SBP and SP produced a pronounced mucosal anti-inflammatory profile by concurrently attenuating pro-inflammatory cytokine transcription and promoting IL-10 expression.

**Fig. 1:**
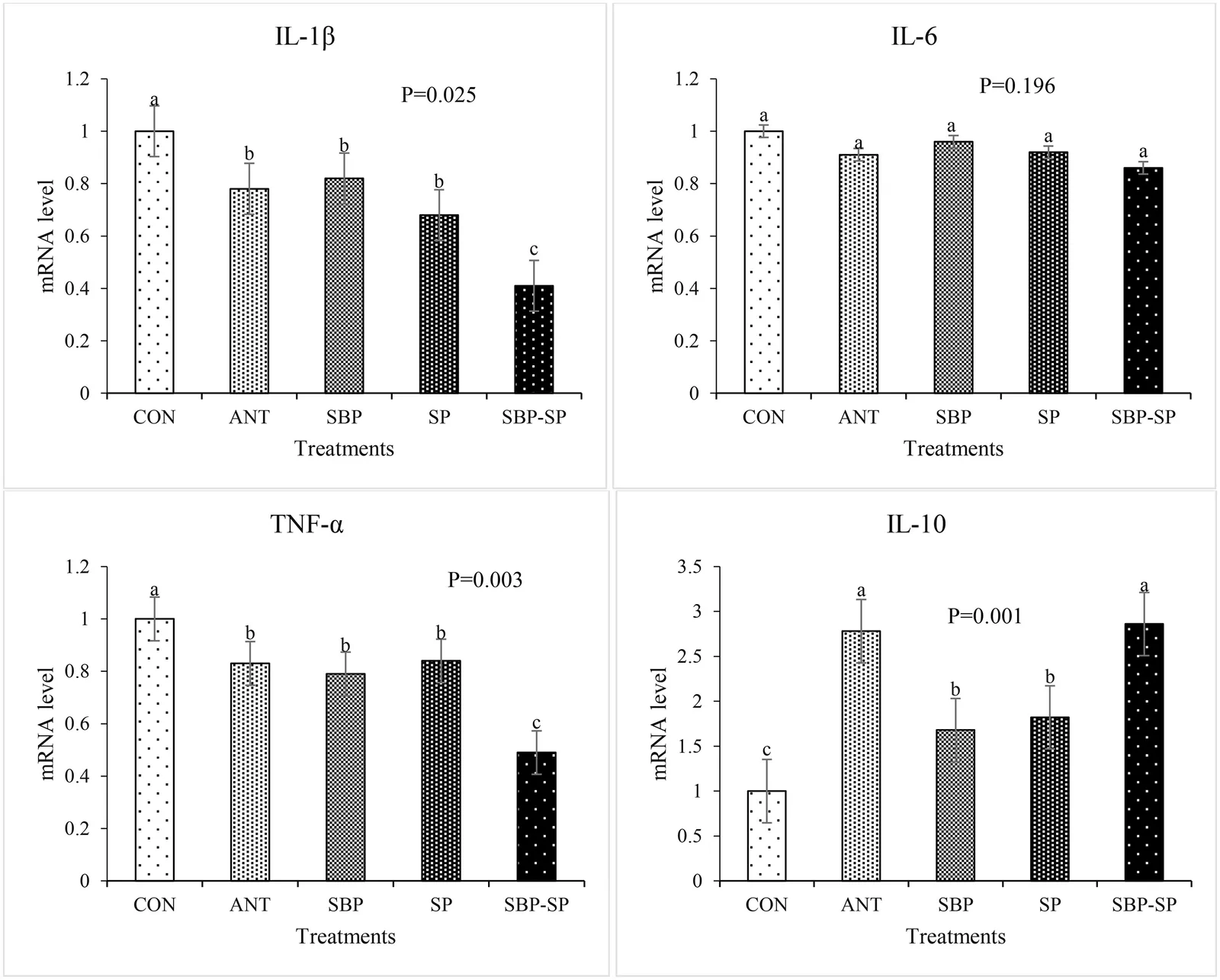
Effects of soybean bioactive peptides and Spirulina on the mRNA expression of inflammatory cytokines in the jejunal mucosa of broiler chickens. CON = basal diet; ANT = basal diet + antibiotic; SBP= basal diet + soybean-derived bioactive peptides; SP = basal diet + Spirulina powder; SBP-SP = basal diet + soybean-derived bioactive peptides + Spirulina powder. ^a-b^ Means within a variable with no common superscript differ significantly (P<0.05).

### Intestinal barrier gene expression

The effects of dietary treatments on the mRNA expression of intestinal barrier integrity markers and mucin secretion in the jejunum are presented in Fig. 2. The expression of *ZO-1* was significantly upregulated in the SBP-SP group compared to the CON group (P = 0.016), while the ANT, SBP, and SP groups showed intermediate levels without significant differences from either the control or the SBP-SP treatment. Furthermore, dietary supplementation significantly influenced the expression of *MUC2* (P = 0.008). The highest mRNA levels of *MUC2* were observed in birds fed the SBP-SP diet, which were significantly greater than those in the CON, SBP, and SP groups. The ANT group also exhibited a significant increase in *MUC2* expression compared to the CON group, yet it remained lower than the SBP-SP treatment. In contrast, the mRNA expression levels of *Occludin* (P = 0.284) and *Claudin-1* (P = 0.315) were not significantly affected by any of the dietary treatments, although a numerical increase was observed in the supplemented groups relative to the control. These results indicate that the co-supplementation of SBP and SP selectively reinforced tight junction (*ZO-1*) and protective mucous layer (*MUC2*) expression in the jejunal epithelium.

**Fig. 2:**
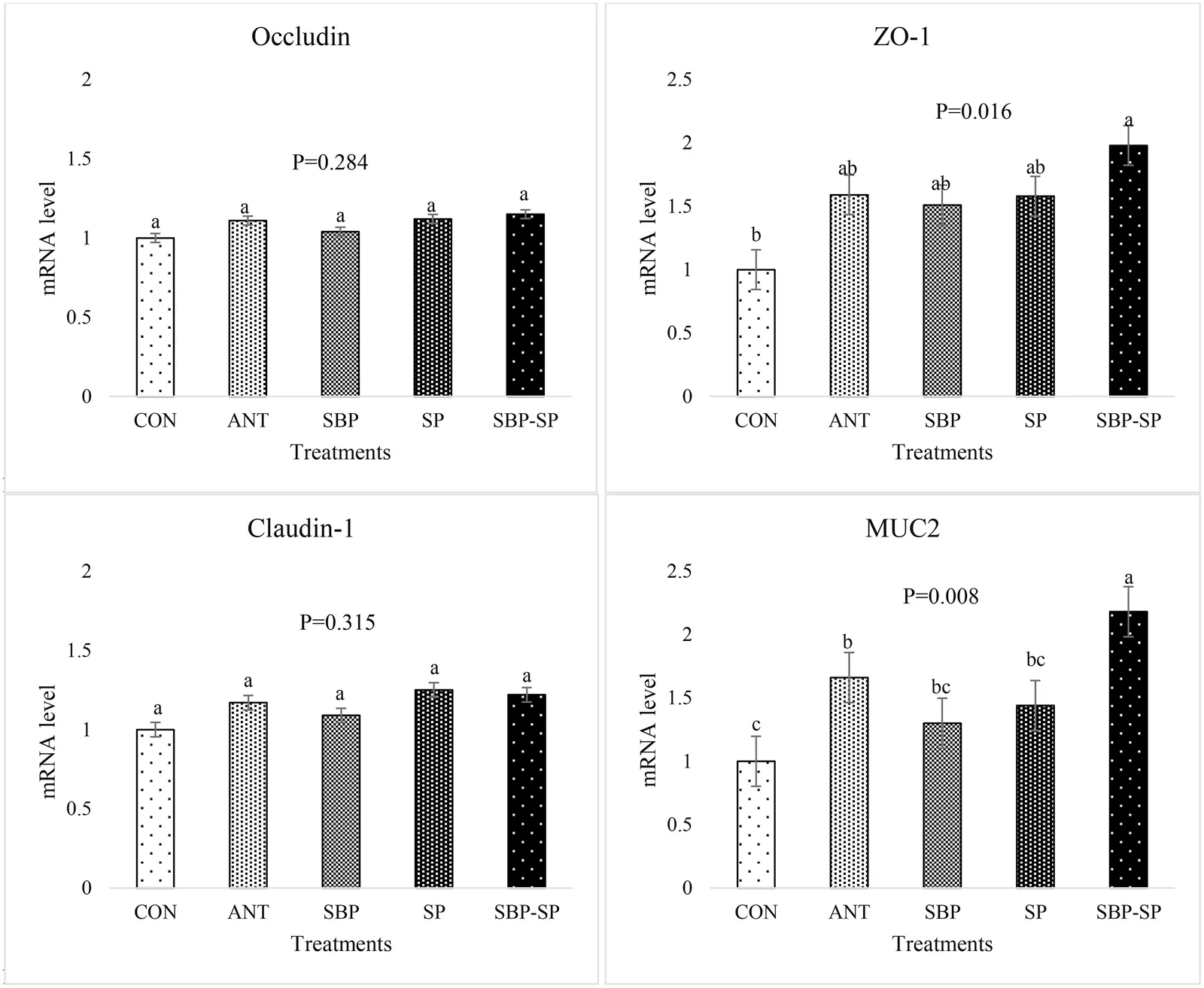
Effects of soybean bioactive peptides and Spirulina on the mRNA expression of tight junction proteins and mucin-2 in the jejunal mucosa of broiler chickens. CON = basal diet; ANT = basal diet + antibiotic; SBP= basal diet + soybean-derived bioactive peptides; SP = basal diet + Spirulina powder; SBP-SP = basal diet + soybean-derived bioactive peptides + Spirulina powder. ^a-b^ Means within a variable with no common superscript differ significantly (P<0.05).

### Nutrient transporter gene expression

The mRNA expression of key nutrient transporters in the jejunal mucosa is presented in Fig. 3. The mRNA levels of *SLC15A1* (also known as *PepT1*) were significantly upregulated in broilers fed the SBP and SBP-SP diets compared to the CON, ANT, and SP groups (P = 0.002). Similarly, the expression of *SLC6A19* was markedly increased in the SBP and SBP-SP treatments compared with the other groups (P = 0.001), with the lowest expression observed in the CON group. Regarding cationic amino acid transport, the mRNA expression of *SLC7A1* was significantly higher in birds receiving SBP and SBP-SP than those in the CON group (P = 0.027), while ANT and SP showed intermediate values. In contrast, the mRNA expression of *SLC3A1* was not significantly affected by any of the experimental diets (P = 0.516), although all supplemented groups showed a slight numerical increase compared to the control. Altogether, dietary SBP, whether provided alone or in combination with SP, was the determining factor upregulating the mucosal expression of peptide and neutral/cationic amino acid transporters.

**Fig. 3:**
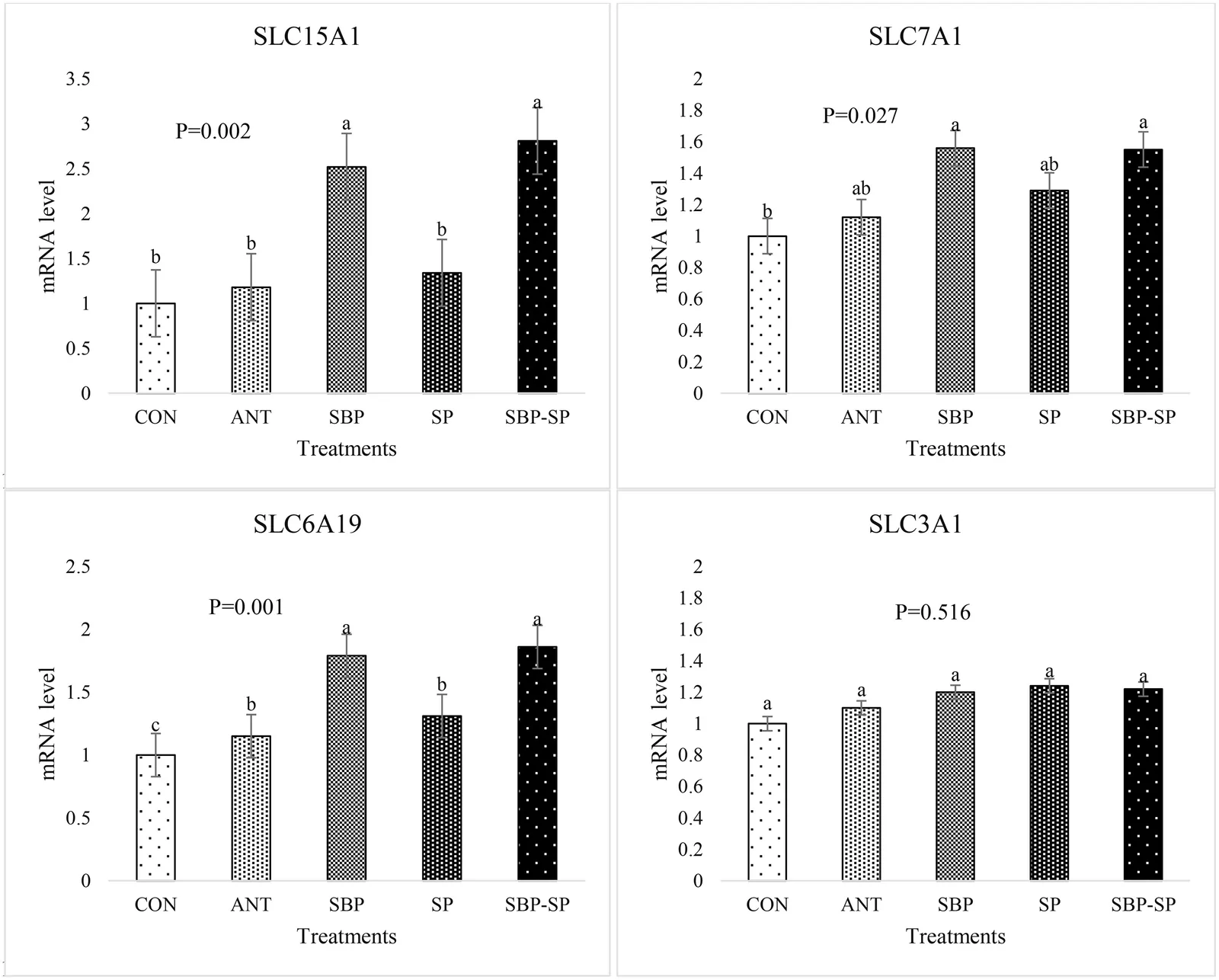
Effects of soybean bioactive peptides and Spirulina on the mRNA expression of nutrient transporters in the jejunal mucosa of broiler chickens. CON = basal diet; ANT = basal diet + antibiotic; SBP= basal diet + soybean-derived bioactive peptides; SP = basal diet + Spirulina powder; SBP-SP = basal diet + soybean-derived bioactive peptides + Spirulina powder. ^a-b^ Means within a variable with no common superscript differ significantly (P<0.05).

## Discussion

### Growth performance

The results of the present study indicated that dietary supplementation with SBP and SP, particularly in combination (SBP-SP), significantly improved the growth performance of broiler chickens. In agreement with our findings, Zhang et al. (2026) reported that the inclusion of bioactive peptides in broiler diets enhanced BWG and FCR by improving protein utilization. Similarly, the growth-promoting effects of SP observed in the SBP-SP and SP groups are consistent with the results of Spínola et al. (2024), who found that SP supplementation at low levels (1–5 g/kg) effectively increases feed intake and weight gain in broiler chickens. However, our results during the finisher phase showed no significant differences among treatments, which contrasts with the study by Alghamdi et al. (2024) and Mohammadrezaeia et al. (2025), where growth stimulation persisted until 42 days. This discrepancy might be due to the maturation of the digestive system in older birds, which potentially reduces their reliance on exogenous bioactive compounds compared to the starter and grower phases. The increase in feed intake and BWG is likely linked to the enhanced digestive enzyme activities (amylase and protease) and the upregulation of nutrient transporters (*SLC15A1* and *SLC6A19*). We found that the SBP-SP diet markedly increased the mRNA expression of these transporters, which facilitates the absorption of di-peptides and amino acids. This is supported by Miraalami et al. (2025), who stated that SBP can directly stimulate the expression of PepT1 in the intestinal mucosa. Furthermore, the superior FCR in the SBP-SP group during the grower phase is highly correlated with the improved intestinal morphology observed in our study. The increase in villus height and VH:CD ratio provided a larger absorptive surface area, which, when combined with the enhanced intestinal barrier function (upregulation of *ZO-1* and *MUC2*), ensures that nutrients are absorbed more efficiently. As noted by Ducatelle et al. (2023), a more robust gut barrier reduces the metabolic cost associated with subclinical inflammation. This is further corroborated by our gene expression data, where the SBP-SP treatment suppressed pro-inflammatory cytokines (*IL-1β* and *TNF-α*) and elevated *IL-10*, thereby redirecting energy from immune-system activation toward tissue accretion and growth (Wan et al., 2022).

### Protein and amino acid digestibility

The apparent ileal digestibility of crude protein and major amino acids was significantly enhanced by the dietary supplementation of SBP and SBP-SP, matching the performance of the ANT group. These findings are in line with previous studies reporting that dietary bioactive peptides improve nitrogen retention and amino acid digestibility in broilers (Abdollahi et al., 2017; Qazi et al., 2025; Rezaei et al., 2025). The highly digestible nature of SBP, which are already partially hydrolyzed, bypasses the initial energy-consuming steps of protein digestion, leading to rapid absorption in the upper jejunum (Osho et al., 2019). In contrast, birds fed the SP group alone did not show a significant improvement in AA digestibility compared to the CON group. This is consistent with Boney and Moritz (2017), who noted that while SP is rich in protein, its cell wall (composed of complex peptidoglycan) may partially resist endogenous enzyme degradation. In our study, protease activity was significantly elevated in all treated groups compared with the CON group. Exogenous bioactive peptides are known to stimulate the secretion of cholecystokinin, which in turn triggers the pancreas to release more digestive enzymes, including trypsin and chymotrypsin (Song et al., 2025). This elevated protease pool in the lumen ensures a more thorough breakdown of both dietary and microbial proteins into absorbable peptides and free amino acids (Borda-Molina et al., 2019). More importantly, the digestive efficiency in the SBP and SBP-SP groups is directly corroborated by our nutrient transporter gene expression data. The significant upregulation of *SLC15A1* (*PepT1*) in the SBP and SBP-SP groups explains the enhanced digestibility of CP and total amino acids. PepT1 is a high-capacity, low-affinity transporter that absorbs di- and tri-peptides more rapidly and with lower energy expenditure than free amino acid transporters (Miska and Fetterer, 2017).

Furthermore, the increased digestibility of essential neutral amino acids (such as Met, Ile, and Leu) and non-essential amino acids (such as Asp and Glu) in the SBP-SP and SBP groups is strongly supported by the upregulation of *SLC6A19* (*B0AT1*). *B0AT1* is the primary transporter responsible for the sodium-dependent absorption of neutral amino acids in the brush border membrane (Adhikari et al., 2022). Additionally, the improved digestibility of Lys and Arg (basic amino acids) in these groups matches the elevated expression of *SLC7A1* (*CAT1*), which specifically facilitates the transport of cationic amino acids (Khwatenge et al., 2020). On the other hand, the significantly higher villus height and VH:CD ratio in these groups maximized the physical surface area available for transporter insertion and nutrient contact. This structural adaptation, combined with the reduction in jejunal inflammation (downregulation of *IL-1β* and *TNF-α*), maintained the integrity of the brush border membrane, preventing enterocyte turnover and ensuring that the newly expressed transporters were fully functional. The synergistic action of SBP and SP in the SBP-SP group therefore represents a complete optimization of the digestive-absorptive axis: SBP drives the enzymatic and molecular machinery (enzymes and transporters), while SP supports gut health and mucosal protection (via antioxidant and anti-inflammatory pathways), together culminating in maximum amino acid bioavailability.

### Digestive enzyme activities

The present study demonstrated that dietary supplementation with SBP, SP, and their combination significantly enhanced the activities of amylase and total protease in broiler chickens compared with the control group. These results are consistent with previous reports indicating that functional feed additives such as bioactive peptides can stimulate pancreatic enzyme secretion and improve digestive efficiency in broiler chickens (Mokhtari et al., 2024; Rezaei et al., 2025). Similarly, Park et al. (2018) and Alghamdi et al. (2024) reported that dietary SP supplementation increased digestive enzyme activity in broilers, potentially through its rich supply of bioactive compounds, vitamins, and pigments that support digestive physiology. The improvement in protease activity observed in the SBP and SBP-SP groups can be attributed to the biological properties of SBP (Guo et al., 2021). Short-chain peptides are known to stimulate gastrointestinal hormonal responses, particularly the secretion of cholecystokinin, which enhances pancreatic enzyme release, including trypsin and chymotrypsin (Xie et al., 2025). This mechanism has been documented in studies where dietary peptides increased pancreatic secretory activity and improved protein hydrolysis in the small intestine (Rezaei et al., 2025). The higher protease activity observed in the SBP-SP group is therefore consistent with the improved apparent ileal digestibility of crude protein and several amino acids reported in present study, suggesting a strong functional link between enzyme secretion and nutrient utilization. In addition, the elevated amylase activity in the SP and SBP-SP groups may be associated with the high content of bioactive compounds present in SP, including phycocyanin, polysaccharides, and essential micronutrients, which have been reported to enhance digestive gland function and intestinal metabolism (Abdel-Wahab et al., 2023). Enhanced amylase secretion likely improves carbohydrate digestion, thereby providing additional metabolizable energy that can support growth performance (Ma et al., 2020).

### Jejunum morphology

Intestinal morphology is widely regarded as a key indicator of gut health and nutrient absorption efficiency in broiler chickens (Liao et al., 2020). In the present study, dietary supplementation with SBP-SP and ANT significantly increased villus height and the villus height to crypt depth ratio (VH:CD) in the jejunum compared with the CON group. These findings are consistent with previous studies demonstrating that functional feed additives, including bioactive peptides and microalgae, can improve intestinal architecture and enhance absorptive capacity in poultry (Mamashli and Ghorbani, 2025; Zhang et al., 2026). Increased villus height generally reflects a greater epithelial surface area for nutrient absorption, whereas a higher VH:CD ratio indicates improved intestinal maturity and reduced epithelial turnover (Alagbe et al., 2026). The substantial improvement in villus surface area observed in the SBP-SP and ANT groups further confirms the beneficial effects of these treatments on intestinal development. Larger villus surface areas provide more space for the localization of digestive enzymes and nutrient transporters along the brush border membrane (Sun et al., 2026). This structural adaptation may partly explain the improved amino acid digestibility reported in present study and the enhanced expression of nutrient transporter genes (*SLC15A1, SLC6A19*, and *SLC7A1*) shown in Figure 3. Similar associations between improved villus architecture and nutrient transporter activity have been reported in broiler studies where dietary bioactive compounds stimulated both intestinal morphology and absorptive gene expression (Hakim et al., 2022). The positive effect of the SBP-SP combination on intestinal morphology may also be attributed to the complementary biological functions of its components. SBP are known to stimulate intestinal cell proliferation and enhance mucosal repair (Peng et al., 2025), while SP provides potent antioxidant and anti-inflammatory compounds, such as phycocyanin and carotenoids, which protect intestinal epithelial cells from oxidative stress (Khan et al., 2020). The reduced expression of pro-inflammatory cytokines (*IL‑1β* and *TNF‑α*) and the increased expression of *IL‑10* observed in the SBP-SP group likely contributed to maintaining intestinal epithelial integrity and promoting villus development.

### Immune response

The humoral immune response plays a crucial role in protecting broiler chickens against viral infections and is often used as an indicator of immunocompetence in poultry nutrition studies. In the present study, dietary supplementation with SP and the SBP-SP combination significantly enhanced antibody titers against IBV at both 21 and 42 days of age, as well as against IBDV at 21 days. These findings are consistent with previous studies reporting that SP supplementation can stimulate humoral immunity and increase antibody production in broiler chickens (Feshanghchi et al., 2022). The immunostimulatory properties of SP have been largely attributed to its rich content of phycocyanin, polysaccharides, vitamins, and carotenoids, which are known to enhance macrophage activation and lymphocyte proliferation (El-Shall et al., 2023). The stronger immune response observed in the SP and SBP-SP groups may also be associated with the antioxidant properties of SP. Oxidative stress is known to impair immune cell function and antibody synthesis, whereas improved antioxidant defense supports optimal immune responses (Salah et al., 2025). In the current study, birds receiving SP and SBP-SP exhibited enhanced antioxidant status, as indicated by higher SOD, GPx, CAT, and TAC levels and reduced MDA concentrations. Similar relationships between improved antioxidant capacity and enhanced humoral immunity have been reported in broilers supplemented with microalgae or plant-derived antioxidants (Mirzaie et al., 2017). Interestingly, the SBP and ANT treatments did not significantly increase antibody titers compared with the control group. This observation suggests that although bioactive peptides and antibiotics may improve growth performance and nutrient utilization, their direct stimulatory effects on humoral immunity may be limited compared with SP -derived bioactive compounds.

### Immune organ indices

The development of primary and secondary lymphoid organs provides important insights into the immune status of broiler chickens. Birds receiving SBP-SP, SP, and ANT diets exhibited significantly higher bursal indices compared with those in the CON and SBP groups. The bursa of Fabricius is the primary lymphoid organ responsible for B lymphocyte differentiation and antibody production in birds, and an increase in its relative weight is often associated with enhanced humoral immune capacity (Cazaban et al., 2015). Similarly, the Thymus index was significantly increased in the SP and SBP-SP groups compared with the CON and SBP treatments. The thymus is essential for T-cell maturation and cellular immune responses, and its development is closely related to the immune competence of young birds. Previous studies have reported that SP supplementation promotes lymphoid organ development by stimulating immune cell proliferation and improving antioxidant protection within immune tissues (Fu et al., 2022). The enhanced thymus development observed in the SBP-SP group therefore supports the improved immune responsiveness detected in the antibody titer results. Interestingly, although the ANT group showed an increased bursal index, its thymus index was intermediate and did not reach the levels observed in the SP and SBP-SP groups. This suggests that while antibiotics may influence immune organ development indirectly through gut microbial modulation (Zhao et al., 2024), they may not provide the same immunostimulatory effects as bioactive compounds derived from microalgae.

### Antioxidant status

In the present study, dietary supplementation with SBP, SP, and their combination significantly improved the antioxidant status of broiler chickens. This was evidenced by a significant reduction in serum MDA levels in the SBP, SP, and SBP-SP groups compared with the CON and ANT groups, indicating reduced lipid peroxidation and oxidative damage. Similar antioxidant effects of bioactive peptides and microalgae have been reported in previous poultry studies (Omar et al., 2022; Peng et al., 2024). At the same time, antioxidant defenses were enhanced, as shown by increased activities of SOD, GPx, and CAT, along with higher TAC in birds receiving SP and SBP-SP diets. SP is rich in antioxidant compounds such as phycocyanin, carotenoids, and phenolic substances, which can enhance endogenous antioxidant enzyme systems and improve redox balance (Salah et al., 2025). In addition, SBP have been reported to possess free radical scavenging activity and may stimulate antioxidant enzyme expression (Saadoon and Abbas, 2024). The superior antioxidant status observed in the SBP-SP group likely reflects a synergistic interaction between SBP and SP-derived antioxidants. While SBP contribute free radical scavenging activity and may stimulate antioxidant enzyme expression (Wei et al., 2024), SP provides a diverse array of natural antioxidant molecules that reinforce cellular defense systems (Moustafa et al., 2021). This combined effect not only reduces oxidative damage but may also support improved intestinal integrity, immune function, and growth performance observed in this study.

### Immune gene expression

The present study demonstrated that dietary supplementation with SBP and SP, particularly in combination (SBP-SP), markedly modulated inflammatory signaling in the jejunal mucosa. The significant downregulation of the pro‑inflammatory cytokines *IL‑1β* and *TNF‑α* in the SBP-SP group suggests a substantial attenuation of intestinal inflammation (Liu et al., 2022). Similar anti‑inflammatory effects have been reported for bioactive peptides and SP‑derived compounds, which can regulate immune signaling pathways and reduce excessive cytokine production (Xie et al., 2020; Omar et al., 2022). Concurrently, the marked upregulation of *IL‑10* in the SBP-SP and ANT groups indicates an enhanced anti‑inflammatory response, as *IL‑10* plays a crucial role in suppressing inflammatory reactions and maintaining mucosal immune homeostasis (Rodríguez et al., 2023). The simultaneous reduction of pro‑inflammatory cytokines and increase in *IL‑10* suggests that SBP-SP improved intestinal immune balance, which likely contributed to the improved gut morphology and nutrient absorption observed in this study.

### Intestinal barrier gene expression

The integrity of the intestinal barrier is essential for maintaining gut health and preventing the translocation of pathogens and toxins. In the present study, birds receiving the SBP-SP diet showed a significant upregulation of *ZO‑1* and *MUC2* expression in the jejunal mucosa, indicating an improvement in epithelial barrier function and mucosal protection. *ZO‑1* is a critical component of tight junction complexes that regulate paracellular permeability, while *MUC2* is the primary mucin responsible for forming the protective mucus layer covering the intestinal epithelium (Yosi and Metzler-Zebeli, 2023). The elevated expression of these genes suggests that the SBP-SP treatment strengthened the physical and chemical defenses of the intestinal barrier. These findings are consistent with previous studies reporting that bioactive peptides and microalgal compounds can enhance tight junction integrity and stimulate mucin secretion, thereby protecting intestinal epithelial cells from inflammatory damage (Xie et al., 2020; Peng et al., 2025). This may be due to the broad-spectrum antibacterial properties of bioactive peptides, which can effectively inhibit harmful bacteria and upregulate the expression of tight-junction protein in intestinal epithelium (Segawa et al., 2011; Miyauchi et al., 2012).

### Nutrient transporter gene expression

The upregulation of nutrient transporter genes in the jejunum provides an important molecular explanation for the improved protein and amino acid utilization observed in the SBP and SBP-SP groups. As the intestine is the major site of nutrient absorption, the expression abundance of peptide and amino acid transporters directly determines the efficiency of nutrient assimilation. In the present study, *SLC15A1* (*PepT1*) was significantly upregulated in broilers fed SBP and SBP-SP diets, indicating an enhanced capacity for the transmembrane transport of di‑ and tri‑peptides. This finding agrees with Zhang et al. (2026), who reported that bioactive peptides and free amino acids generated during microbial–enzymatic fermentation of feather meal elevated intestinal peptide/amino acid transporter expression in broilers. Similarly, *PepT1* has been described as the predominant transporter responsible for oligopeptide uptake, with its expression level positively correlated with intestinal peptide absorption efficiency (Adibi, 2003). Therefore, the increased *PepT1* expression in the SBP and SBP-SP groups likely facilitated the absorption of small peptides derived from SBP, contributing to the improved crude protein and amino acid digestibility reported in present study. In addition to peptide transport, the expression of amino acid transporters was also enhanced by SBP-containing diets. The marked upregulation of *SLC6A19* (*B0AT1*) in the SBP and SBP-SP groups suggests an improved capacity for the absorption of neutral amino acids, including methionine, leucine, isoleucine, and threonine, whose digestibility was significantly increased in these treatments (Sadr et al., 2025). Moreover, the higher expression of *SLC7A1* (*CAT1*) supports enhanced transport of cationic amino acids, such as lysine and arginine (Frazier et al., 2008), which also showed improved ileal digestibility in birds receiving SBP-SP and SBP diets. These results are consistent with the concept proposed by Zhang et al. (2026) that small‑molecular‑weight nutrients, including bioactive peptides and free amino acids, can initiate the transcription of transporter proteins, thereby enhancing intestinal nutrient assimilation. In that study, transporter upregulation was accompanied by activation of the mTOR/S6K1 nutrient‑sensing pathway, suggesting that peptide- and amino acid-rich substrates may improve nutrient utilization through coordinated regulation of transporter expression and cellular nutrient signaling.

## Conclusion

In conclusion, dietary supplementation with bioactive peptides derived from soybean meal (SBP) and *Spirulina* powder (SP), particularly in combination at 1 g/kg SBP and 1 g/kg SP (SBP-SP), increased ADG and reduced FCR in broiler chickens compared to individual supplementation at 2 g/kg. The SBP-SP treatment enhanced protein and amino acid digestibility, stimulated digestive enzyme activities, and improved jejunal morphology and intestinal barrier integrity. These effects were accompanied by increased antioxidant capacity, modulation of immune responses through reduced pro-inflammatory cytokine expression and increased *IL-10* expression, and upregulation of key nutrient transporter genes involved in peptide and amino acid absorption. Collectively, these findings indicate that the combined inclusion of soybean meal-derived bioactive peptides (1 g/kg) and *Spirulina* powder (1 g/kg) exerts synergistic effects on digestive efficiency, intestinal function, and physiological status, demonstrating that this specific combination serves as an effective natural alternative to antibiotic growth promoters (avilamycin at 100 g/ton) in broiler nutrition.

## Author contributions

Ali Jafarzadeh contributed substantially to the acquisition and analysis of data and the execution of the experiments, drafted the original manuscript, reviewed and explicitly approved the final version, and agrees to be personally accountable for the accuracy and integrity of the work. Kaveh Jafari-Khorshidi made substantial contributions to the conception and design of the work as well as the interpretation of data, provided critical intellectual revisions to the manuscript, reviewed and explicitly approved the final version, and agrees to be personally accountable for the accuracy and integrity of the work. Mohammad Ali Jafari contributed substantially to the conception and design of the research and the supervision of project implementation, reviewed and explicitly approved the final version of the manuscript, and agrees to be personally accountable for the accuracy and integrity of the work. Shahabodin Gharahveysi contributed to the execution of experiments, data processing, and interpretation of results, reviewed and explicitly approved the final version of the manuscript, and agrees to be personally accountable for the accuracy and integrity of the work. Sakineh Asadzadeh developed the research concept, provided study resources, supervised the entire research process, performed the final critical revision of the manuscript, reviewed and explicitly approved the final version, and agrees to be personally accountable for the accuracy and integrity of the work.

## Disclosures

The authors declare that they have no known competing financial interests or personal relationships that could have appeared to influence the work reported in this paper.
